# Using Colistin as a Trojan Horse: Inactivation of Gram-Negative Bacteria with Chlorophyllin

**DOI:** 10.3390/antibiotics8040158

**Published:** 2019-09-20

**Authors:** Peter Richter, Marcus Krüger, Binod Prasad, Susanne Gastiger, Mona Bodenschatz, Florian Wieder, Andreas Burkovski, Walter Geißdörfer, Michael Lebert, Sebastian M. Strauch

**Affiliations:** 1 Cell Biology Division, Department of Biology, Friedrich-Alexander University Erlangen-Nuremberg, Staudtstraße 5, 91058 Erlangen, Germany; peter.richter@fau.de (P.R.); flo_20@hotmail.com (F.W.); michael.lebert@fau.de (M.L.); 2 Clinic for Plastic, Aesthetic and Hand Surgery, Otto von Guericke University Magdeburg, Leipziger Str. 44, 39120 Magdeburg, Germany; 3 Microbiology Division, Department of Biology, Friedrich-Alexander University Erlangen-Nuremberg, Staudtstraße 5, 91058 Erlangen, Germany; susanne.gastiger@fau.de (S.G.); mona.bodenschatz@fau.de (M.B.); andreas.burkovski@fau.de (A.B.); 4 Microbiological Diagnostics, Clinical Microbiology, Immunology and Hygiene, University Hospital Erlangen, Wasserturmstraße 3/5, 91054 Erlangen, Germany; walter.geissdoerfer@uk-erlangen.de; 5 Postgraduate Program in Health and Environment, University of Joinville Region, Rua Paulo Malschitzki, 10, Joinville 89219-710, Brazil; sebastian.michael@univille.br

**Keywords:** chlorophyll, bacteria, photosensitization, combination therapy, antibiotic resistance, *mcr-1*

## Abstract

Colistin (polymyxin E) is a membrane-destabilizing antibiotic used against Gram-negative bacteria. We have recently reported that the outer membrane prevents the uptake of antibacterial chlorophyllin into Gram-negative cells. In this study, we used sub-toxic concentrations of colistin to weaken this barrier for a combination treatment of *Escherichia coli* and *Salmonella enterica* serovar Typhimurium with chlorophyllin. In the presence of 0.25 µg/mL colistin, chlorophyllin was able to inactivate both bacteria strains at concentrations of 5–10 mg/L for *E. coli* and 0.5–1 mg/L for *S.* Typhimurium, which showed a higher overall susceptibility to chlorophyllin treatment. In accordance with a previous study, chlorophyllin has proven antibacterial activity both as a photosensitizer, illuminated with 12 mW/cm^2^, and in darkness. Our data clearly confirmed the relevance of the outer membrane in protection against xenobiotics. Combination treatment with colistin broadens chlorophyllin’s application spectrum against Gram-negatives and gives rise to the assumption that chlorophyllin together with cell membrane-destabilizing substances may become a promising approach in bacteria control. Furthermore, we demonstrated that colistin acts as a door opener even for the photodynamic inactivation of colistin-resistant (*mcr-1*-positive) *E. coli* cells by chlorophyllin, which could help us to overcome this antimicrobial resistance.

## 1. Introduction

After the discovery of the first antibiotics, penicillin in 1929 and streptomycin in 1943, scientists prophesized the end of infectious diseases that had plagued humankind over centuries. Twenty novel classes of antibiotics were launched during the ‘Golden Age’ of antibiotic discovery between 1940 and 1962, one-half of the drugs commonly used today [1,2]. Unfortunately, the widespread and improper use of antibiotics has strongly contributed to the emergence and spread of antibiotic resistance among bacteria that are difficult to treat or—in case of multidrug-resistant (MDR) pathogens—may even be untreatable with conventional drugs [3,4]. MDR patterns in Gram-positives and Gram-negatives have cast a shadow on the assumed victory over pathogenic bacteria, undermining every clinical and public health program designed to control infectious diseases worldwide. Especially Gram-negative bacteria including most of the ‘ESKAPE pathogens’ (*Enterococcus faecium*, *Staphylococcus aureus*, *Klebsiella pneumoniae*, *Acinetobacter baumannii*, *Pseudomonas aeruginosa*, and *Enterobacter* species) [5] represent a major threat (World Health Organization 2017) as they are responsible for a wide spectrum of diseases (viz. urinary tract, bloodstream, airway, sexually transmitted, and health care-associated infections). Recently MDR Gram-negatives have been isolated from humans with increasing frequency. In the world’s richest countries, the massive use of antibiotics (also in hospitals) offers breeding grounds for drug-resistant ‘superbugs’ causing deadly infections [6,7] and enables MDRs to spread easily. However, developing countries are also burdened by antibiotic misconduct: limited access to medical care with effective treatments, inadequate patient education leading to inappropriate self-medication, unauthorized sale of antibiotics, the availability of counterfeit medicines as well as non-human use of antibiotics such as in animal production exacerbated antimicrobial resistance [8,9,10,11].

**Figure 1:**
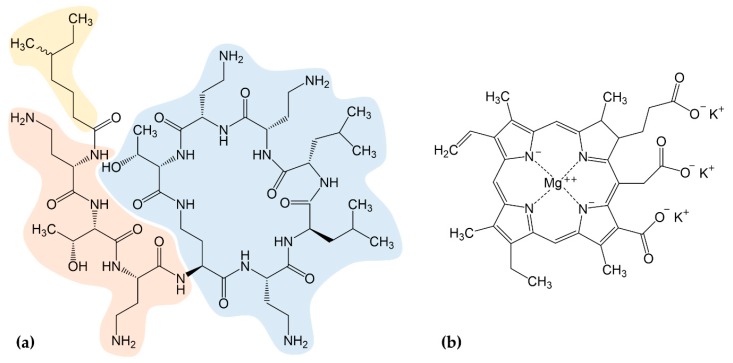
(**a**) The chemical structure of colistin is composed of three parts: a hydrophobic fatty-acyl tail (yellow), a linear tripeptide segment (orange), and a hydrophilic, heptapeptide ring (blue); (**b**) Chemical structure of chlorophyllin. A combination of both components was used to control Gram-negative bacteria.

Hospital- and community-acquired infections are emerging, and as traditional antibiotics are becoming less and less effective, we are increasingly relying on our ‘last resort’ antibiotics. Recently, the WHO Essential Medicines List was forced to classify antibiotics into three groups—AWaRe: access, watch, and reserve—and thus specifies which antibiotics to use for infections [12]. First bacteria strains resistant against reserve antibiotics were already discovered: In 2008, a carbapenem-resistant (*ndm-1* (New Delhi metallo-β-lactamase 1)-positive) *K. pneumoniae* was found in India [13], and in 2015, a colistin-resistant (*mcr-1* (mobilized colistin resistance 1)-positive) *Escherichia coli* was isolated in China [14]. Colistin (polymyxin E) is a cationic polypeptide (Figure 1a), produced by *Paenibacillus polymyxa* [15] and considered one of the last-resort antibiotics against MDR Gram-negative bacteria such as *E. coli*, *P. aeruginosa*, *K. pneumoniae*, and *A. baumannii* responsible for pneumonia, bacteremia, and urinary tract infections [16,17]. Although development of resistance to colistin is unusual, a few studies have shown that the use of colistin to treat *A. baumannii* and *K. pneumoniae* infections has led to the development of resistant bacterial strains [18,19,20,21,22,23]. Some of them have suggested that heteroresistance to colistin is not as common in MDR *P. aeruginosa* as in MDR *A. baumanii*, but a recent study [24] showed the prevalence and mechanisms of heteroresistance in clinical isolates of the pathogens *E. coli*, *Salmonella enterica*, *K. pneumoniae*, and *A. baumannii* against 28 different antibiotics. In addition, to date, nine homologous plasmid-borne *mcr* genes (*mcr-1* to *-9*) have been described [25]. Especially these genes represent a major threat to public health as they can easily be transmitted via horizontal gene transfer. Facilitated by international traveling and trade, resistance genes have already started to disseminate across the world [26,27]. Within few months after the first description of the *mcr-1* gene, plasmids carrying *mcr-1* were ubiquitously identified in various *Enterobacteriaceae* isolates from animals, food, the environment and humans in over 40 countries worldwide [28,29,30,31,32]. Several other homologous *mcr* genes were subsequently identified in Asia, North America, South America, and European countries including Belgium, Italy, Spain, and Germany [33,34,35,36,37,38,39,40,41]. Today’s main problems comprise a shortage of effective therapies, lack of prevention and control strategies [42], and only a neglectable number of new antibiotics [43]. Scientists urgently need to develop novel treatment options and alternative antimicrobial therapies since we may find ourselves back in the dark, pre-antibiotic ages of medicine.

Antimicrobial treatment with chlorophyll or its derivatives is a very promising approach to control bacteria without the use of conventional antibiotics [44,45,46,47,48,49]. Chlorophyll derivatives have been shown to effectively destroy harmful pest organisms such as mosquito larvae [50], fish ectoparasites [51], *Schistosoma*-transmitting snails [52], human parasites or their larvae [53], as well as human cancer cells [54,55]. In light, they exert strong photodynamic properties (photosensitizers) without the formation of toxic byproducts [56]. The molecules undergo photodynamic reactions in which target structures are destroyed, e.g., via generation of reactive oxygen species (ROS) [57,58,59]. In a recent study, we determined the effect of water-soluble chlorophyllin (Figure 1b) on Gram-positive and Gram-negative model strains [60]. Interestingly, we found that chlorophyllin affects the growth of bacteria also in the absence of light, which indicates a second, light-independent mechanism of action [60]. However, both effects were lower against Gram-negative (*E. coli*) compared to activity against Gram-positive bacteria (*Bacillus subtilis*), which is consistent with earlier photodynamic experiments using chlorophyllin and different bacteria [44,46,47]. The outer membrane of Gram-negatives is known to act as a barrier against various hydrophobic and large hydrophilic substances [61]. It impairs the effects of antibiotics such as novobiocin, rifamycin, lincomycin, clindamycin or fusidic acid which eliminate Gram-positive bacteria very effectively [62] but also protects cells against the uptake of chlorophyllin. This was confirmed by a membrane-deficient strain of *E. coli*, which had been more sensitive against chlorophyllin treatment compared to the wild type [60].

To broaden the application spectrum of chlorophyllin against Gram-negatives, we examined the effects of a combination treatment of chlorophyllin and a substance destabilizing the outer membrane. In this study, we chose a sub-toxic concentration of colistin that penetrates and disrupts the outer membrane through a detergent-like mechanism [63,64,65]. Colistin’s stability against photodegradation makes it suitable for photodynamic experiments [66]. These were performed with *E. coli* DH5α and *Salmonella enterica* serovar Typhimurium. In addition, we tested a *mcr-1*-positive *E. coli* DH5α strain to investigate the possibility of a colistin/chlorophyllin combination therapy to overcome the spreading colistin resistance. Plasmid-borne colistin resistance mediated by *mcr-1* is caused by modification of lipid A resulting in a reduction of polymyxin affinity [14].

## 2. Results

### 2.1. Synergistic Effects of Chlorophyllin in Combination with Colistin on the Growth of E. coli

**Figure 2:**
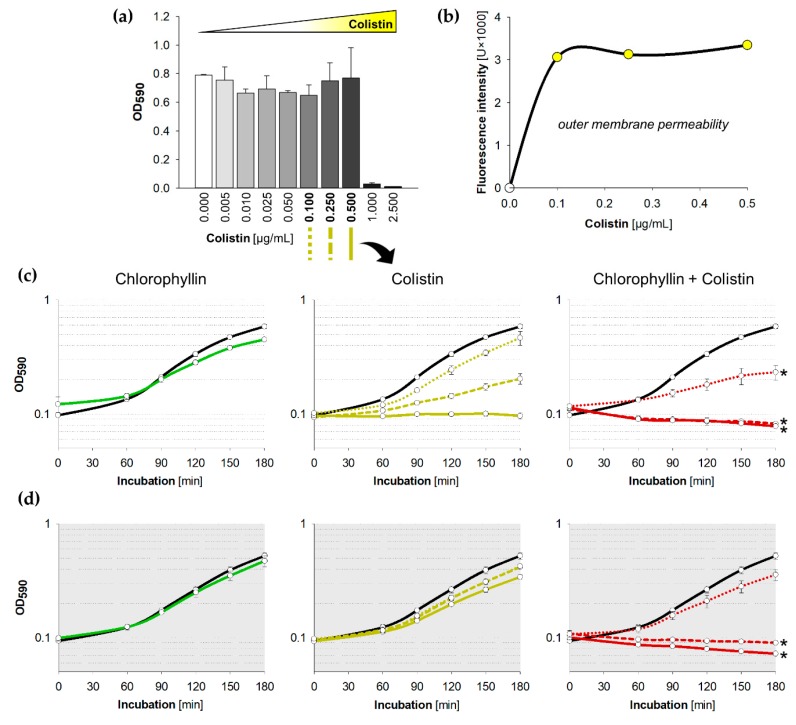
Influence of colistin and chlorophyllin on the growth of *Escherichia coli* DH5α; (**a**) Growth status of *E. coli* cultures in lysogeny broth (LB) supplemented with different colistin concentrations after 24 h incubation at 37 °C; (**b**) outer membrane permeability of *E. coli* in the presence of different colistin concentrations determined by using the NPN uptake assay; (**c**,**d**) Growth pattern of *E. coli* in the presence of 10 mg/L chlorophyllin (green line, first column), colistin (yellow lines, central column) and of a combination of both substances (red lines, right column). The black lines describe growth in LB without any supplementation; (**c**) cells were illuminated with a light intensity of 12 mW/cm^2^; (**d**) cells were incubated in dark. In columns two and three, dotted lines describe growth pattern of cultures in LB + 0.10 µg/mL colistin, dashed lines growth pattern of cultures in LB + 0.25 µg/mL colistin, and solid lines growth pattern of cultures in LB + 0.5 µg/mL colistin. Depicted are measured values (circles) and fitted curves (lines) ± standard deviations (n = 3) showing one representative of three independent experiments. *: *p* < 0.05 vs. chlorophyllin-free samples.

Colistin interacts with lipopolysaccharides (LPS) via replacement of bivalent cations (Mg^2+^, Ca^2+^) stabilizing the LPS that subsequently causes disorganization of the cell membrane. That in turn leads to increased permeability and—in higher concentrations—to loss of cytoplasmic content and eventually cell death which explains its bactericidal effect [63,64,65]. In earlier experiments the Gram-negative model strain *E. coli* DH5α showed no growth inhibition in lysogeny broth (LB) medium supplemented with ≤25 mg/L chlorophyllin both in illuminated and in dark conditions [60]. In the current study we tested growth of *E. coli* DH5α in LB containing 10 mg/L chlorophyllin (a concentration which was found to be efficient against Gram-positive bacteria and that was within a range considered harmless to humans [60]) combined with sub-toxic concentrations of colistin. To elucidate the photodynamic effects of chlorophyllin, the experimental samples were incubated in darkness or illuminated with a standardized light intensity of 12 mW/cm^2^ at 37 °C. The minimal inhibitory concentration (MIC) of colistin was previously determined to be 0.75 µg/mL for *E. coli* DH5α using broth microdilution (MICRONAUT MIC-Strip colistin: 1 µg/mL). In our experimental setup, we found a concentration of 1 µg/mL was necessary to inhibit growth (Figure 2a). Colistin concentrations of ≥1.0 µg/mL inhibited growth of *E. coli* within 180 min, whereas lower concentrations (≤0.5 µg/mL) had only a neglectable effect on proliferation (Table S1). Since these concentrations appeared to be less or non-toxic to the strain and the 1-N-phenylnaphthylamine (NPN) uptake assay confirmed an increase of the outer membrane permeability (Figure 2b), we decided to use colistin with 0.50 µg/mL, 0.25 µg/mL, and 0.10 µg/mL in the experiments together with chlorophyllin.

While supplementation of 10 mg/L chlorophyllin alone did not show any densitometrically detectable effect on *E. coli*, combinations with 0.50 µg/mL or 0.25 µg/mL of colistin completely inhibited the growth in the first hour (Figure 2c,d). The efficacy of chlorophyllin against *E. coli* was confirmed both in light and in darkness validating its photodynamic and light-independent mechanisms of action described before [60]. In fact, at lower colistin concentrations (0.10 µg/mL), chlorophyllin delayed cell growth without complete inhibition of the culture (Figure 2, red dotted lines). Interestingly, we observed a possible additive effect of colistin and light: concentrations of 0.25–0.50 µg/mL colistin impaired the growth of *E. coli* cells illuminated with a light intensity of 12 mW/cm^2^ (Figure S1). This was also confirmed by a slight decrease of colistin’s MIC to 0.5 µg/mL in light.

**Figure 3:**
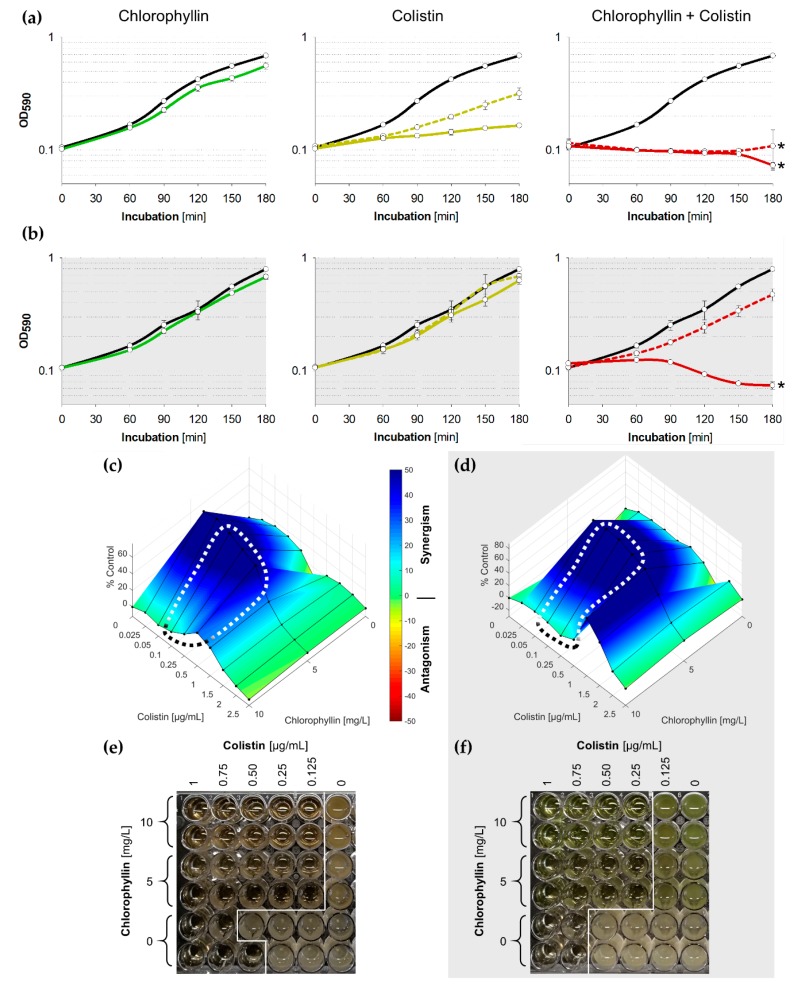
(**a**,**b**) Growth pattern of *E. coli* in the presence of 5 mg/L chlorophyllin (green line, first column), colistin (yellow lines, central column) and of a combination of both substances (red lines, right column). The black lines describe growth in LB without any supplementation; (**a**) cells were illuminated with a light intensity of 12 mW/cm^2^; (**b**) cells were incubated in dark. In columns two and three, dashed lines represent cultures in LB + 0.25 µg/mL colistin, while solid lines are for cultures in LB + 0.5 µg/mL colistin. Depicted are measured values (circles) and fitted curves (lines) ± standard deviations (n = 3) showing one representative of three independent experiments. *: *p* < 0.05 vs. chlorophyllin-free samples; (**c**,**d**) Loewe synergism and antagonism for colistin/chlorophyllin combinations against the growth of *E. coli* as determined by the Combenefit software (Cancer Research UK, Cambridge, UK). A heat map is used to represent the level of synergy (blue color) at each concentration under (**c**) illuminated and (**d**) dark conditions. The area surrounded by dotted line includes the concentration range used for the growth curves. (**e**,**f**) Broth microdilution plates for minimal inhibitory concentration (MIC) determination for colistin/chlorophyllin combinations under (**e**) illuminated and (**f**) dark conditions.

Because experiments revealed a significant inhibitory effect of chlorophyllin/colistin combination treatment on *E. coli*, we decided to use a lower chlorophyllin concentration of 5 mg/L for further experiments (Figure 3). Supplemented with 0.50 µg/mL colistin, chlorophyllin completely inhibited the growth of *E. coli* within three hours both in light and in darkness (Figure 3, red solid lines). In addition, 0.25 µg/mL colistin combined with 5 mg/L chlorophyllin were sufficient to impair cell growth under illumination (Figure 3a, red dashed line). In darkness delayed growth was observed (Figure 3b, red dashed line). Cells were affected by the chlorophyllin/colistin mixture after 90 min in light and 150 min in dark (Figure S2). Based on Combenefit analyses, especially the combination of 5 mg/L chlorophyllin and colistin showed high synergy against the growth of *E. coli* when illuminated (Figure 3c) as well as in darkness (Figure 3d).

To investigate the long-term effect of chlorophyllin and colistin, MICs for colistin/chlorophyllin combinations were determined using broth microdilution. Optical analysis after 24 h confirmed effective combinations of 0.125 µg/mL colistin with 10 or 5 mg/L chlorophyllin when illuminated with 12 mW/cm^2^ (Figure 3e), and effective combinations of 0.25 µg/mL colistin with 10 or 5 mg/L chlorophyllin when incubated in darkness (Figure 3f).

### 2.2. Synergistic Effects of Chlorophyllin in Combination with Colistin on the Growth of S. Typhimurium 

**Figure 4:**
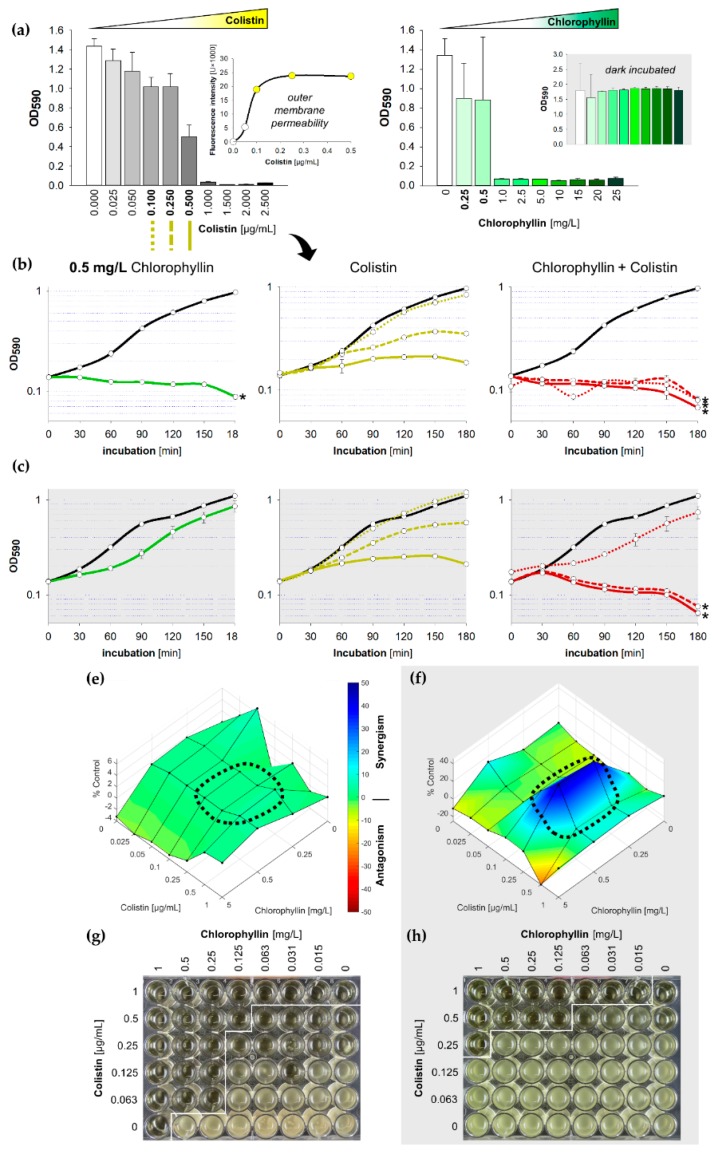
Influence of colistin and chlorophyllin on the growth of *Salmonella enterica* serovar Typhimurium*;* (**a**) Growth status of *S.* Typhimurium cultures in LB supplemented with different concentrations of colistin (left) and chlorophyllin (right) after 24 h incubation. The outer membrane permeability in the presence of different colistin concentrations was determined by using the 1-N-phenylnaphthylamine (NPN) uptake assay; (**b**,**c**) growth pattern of *S.* Typhimurium in the presence of 0.5 mg/L chlorophyllin (green line, first column), colistin (yellow lines, central column) and of a combination of both compounds (red lines, right column). The black lines describe growth in LB without any supplementation; (**b**) cells were illuminated with a light intensity of 12 mW/cm^2^; (**c**) cells were incubated in dark. In columns two and three, dotted lines describe the growth pattern of cultures in LB + 0.10 µg/mL colistin, dashed lines growth pattern of cultures in LB + 0.25 µg/mL colistin, and solid lines growth pattern of cultures in LB + 0.5 µg/mL colistin. Depicted are measured values (circles) and fitted curves (lines) ± standard deviations (n = 3) showing one representative of three independent experiments. *: *p* < 0.05 vs. chlorophyllin-free samples; (**e**,**f**) Loewe synergism and antagonism for colistin/chlorophyllin combinations against the growth of *S.* Typhimurium as determined by the Combenefit software (Cancer Research UK, Cambridge, UK). A heat map is used to represent the level of synergy (blue color) at each concentration under (**e**) illuminated and (**f**) dark conditions. The area surrounded by dotted line includes the concentration range used for the growth curves. (**g**,**h**) Broth microdilution plates for MIC determination for colistin/chlorophyllin combinations under (**g**) illuminated and (**h**) dark conditions.

We further tested the growth of *S.* Typhimurium in LB containing chlorophyllin in combination with colistin. The MIC was determined to be 1 µg/mL colistin for *S.* Typhimurium (Figure 4a). Unexpectedly, the supplementations of ≥1 mg/L chlorophyllin completely inhibited the growth of *Salmonella* cells illuminated with 12 mW/cm^2^ but showed no effect on cells in darkness (Figure 4a).

Under illumination, a concentrations of 0.50 mg/L chlorophyllin (Figure 4b) delayed growth of *Salmonella* in the first three hours to an extent that no synergistic effect of colistin and chlorophyllin could be determined in light. This was further confirmed by Combenefit analyses (Figure 4e). Lower concentration of chlorophyllin (0.25 mg/L) showed similar optical densities but no complete inhibition of the cells were noted as observed in the colony forming unit (CFU) assay (data not shown). In darkness, combinations of 0.25 µg/mL colistin with 0.50 mg/L chlorophyllin were able to prevent cell growth whereas chlorophyllin together with 0.10 µg/mL colistin could only delay proliferation (Figure 4c). Combenefit analyses revealed a high synergism between both substances at a colistin concentration of 0.25 µg/mL (Figure 4f). The NPN uptake assay indicated an increased outer membrane permeability for colistin supplementations of ≥0.1 µg/mL (Figure 4a, small diagram). However, colistin at a concentration of 0.10 µg/mL was insufficient to produce any chlorophyllin-based effects without illumination.

Broth microdilution MIC tests with 24 h incubation displayed effective combinations of 0.063 (0.5) µg/mL colistin with 0.25 (0.125) mg/L chlorophyllin when illuminated with 12 mW/cm^2^ (Figure 4g), and effective combinations of 0.25 (0.5) µg/mL colistin with 1 (0.125) mg/L chlorophyllin when incubated in darkness (Figure 4h).

### 2.3. Synergistic Effects of Chlorophyllin in Combination with Colistin on mcr-1-Positive E. coli

To investigate the possibility of a colistin/chlorophyllin combination therapy to overcome the spreading colistin resistance, we also tested the synergistic effects of colistin/chlorophyllin on a colistin-resistant *E. coli* strain. In contrast to clinical isolates, the generation of a colistin-resistant mutant allows direct comparison of *mcr-1*-positive and *mcr-1*-negative variants of the same strain. Thus, *E. coli* DH5α was transformed with the plasmid pGDP2:*mcr-1* carrying the mobilized colistin resistance gene *mcr-1* [14]. Broth microdilution confirmed that the *mcr-1*-expressing *E. coli* strain showed a 10-times higher MIC (8 µg/mL) for colistin compared to the wild type strain DH5α (0.75 µg/mL).

**Figure 5:**
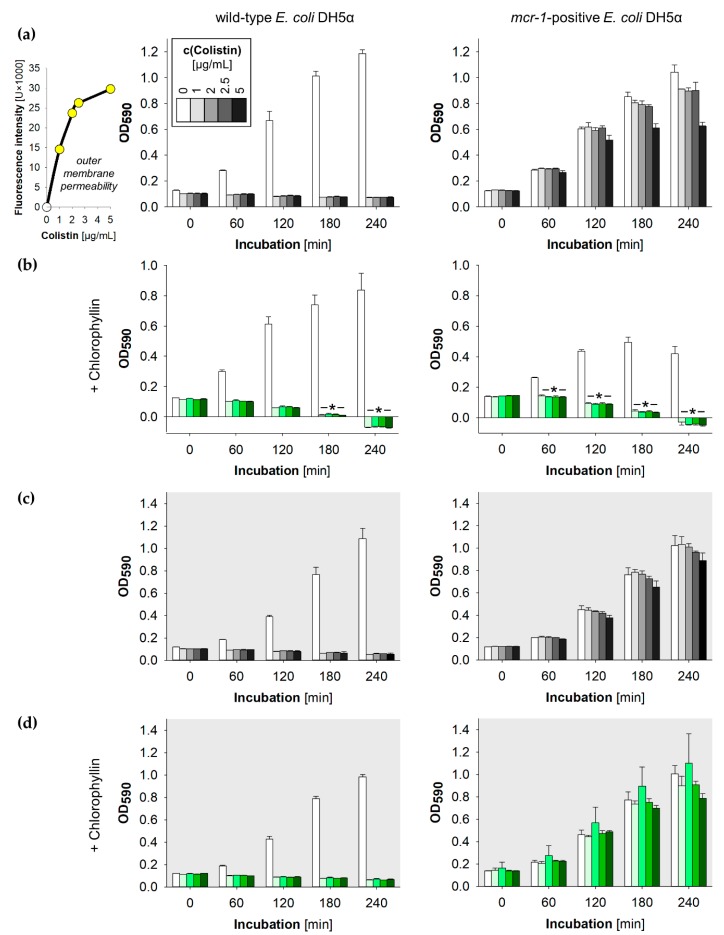
Growth of *E. coli* DH5α (left column) and *mcr-1*-positive *E. coli* DH5α pGDP2:*mcr-1* (right column) supplemented with different colistin concentrations; (**a**,**b**) cells were illuminated with a light intensity of 12 mW/cm^2^ in absence (**a**) and presence (**b**) of 10 mg/L chlorophyllin. The outer membrane permeability was determined by using the NPN uptake assay; (**c**,**d**) cells were incubated protected from light in absence (**c**) and presence (**d**) of 10 mg/L chlorophyllin. Depicted are measured values ± standard deviations (n = 3) showing one representative of three independent experiments. *: *p* < 0.05 vs. chlorophyllin-free samples.

**Figure 6:**
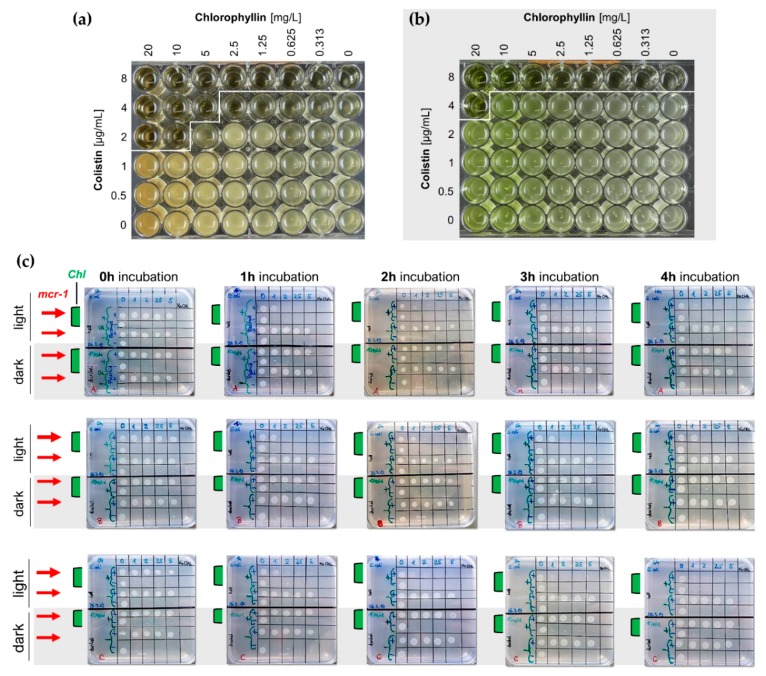
(**a**,**b**) Broth microdilution plates for MIC determination for colistin/chlorophyllin combinations under (**a**) illuminated and (**b**) dark conditions; (**c**) LB agar plates (n = 3) for the evaluation of CFU ability of wild type and *mcr-1*-positive (red arrows) *E. coli* after incubation to chlorophyllin-colistin combinations. Liquid cultures of *E. coli* DH5α were supplemented with 10 mg/L chlorophyllin (Chl, samples with green bars) and different colistin concentrations. Cells grew either illuminated with 12 mW/cm^2^ or protected from light. Samples (2.5 µL) were drawn at different time points (1 h, 2 h, 3 h, 4 h) and transferred onto LB agar plates. After overnight incubation at 37 °C in the dark, colony growth was analyzed.

We noted that the colistin-resistant cells grew profoundly in the presence of all tested colistin concentrations (1.0, 2.0, 2.5, 5.0 µg/mL), while no growth of wild type cells was noted after colistin supplementation (Figure 5a,c). These colistin concentrations further were able to increase the outer membrane permeability of the *mcr-1*-expressing *E. coli* strain (Figure 5a, small diagram). Under illumination, chlorophyllin was able to inhibit the growth of the colistin-resistant *E. coli* in the presence of ≥1.0 µg/mL colistin (Figure 5b). A slightly higher efficiency of chlorophyllin on the *mcr1*-positive strain might be due to membrane effects caused by the kanamycin resistance expressed from the plasmid pGDP2:*mcr-1*. Long-term incubation confirmed effective combinations of 2 (4) µg/mL colistin with 10 (5) mg/L chlorophyllin when illuminated with 12 mW/cm^2^ (Figure 6a), and a possibly effective combination of 4 µg/mL colistin with 20 mg/L chlorophyllin when incubated in darkness (Figure 6b). The results indicate that colistin-resistant cells can be inactivated by the photosensitizer activity of chlorophyllin. This was further affirmed by CFU assays. Viability of *E. coli* cells was determined via colony formation on LB agar plates (Figure 6c). In cultures kept in dark, chlorophyllin supplementation had no effect on the growth of the *mcr-1*-positive *E. coli* strain (Figure 5d).

### 2.4. Confirmation of Chlorophyllin Uptake

To prove that chlorophyllin entrance into *E. coli* and *S.* Typhimurium is facilitated by colistin, we performed fluorescence microscopy with blue light excitation. Figure S3 shows that chlorophyllin (red fluorescence) was clearly accumulated in the cells when *E. coli* DH5α, *mcr-1*-positive *E. coli* DH5α and *S.* Typhimurium were cultured in presence of colistin. This finding confirmed the postulated door-opener ability of colistin for chlorophyllin treatment via membrane destabilization. A weak chlorophyllin fluorescence inside *S.* Typhimurium in absence of colistin could explain the higher susceptibility of *Salmonella* to chlorophyllin treatment.

## 3. Discussion

### 3.1. Strategies to Inactivate Gram-Negative Bacteria with Chlorophyllin

Gram-negative bacteria are not very susceptible to chlorophyllin [26,28,29,42]. In this study, we found that *S.* Typhimurium, against all suspicions, was very sensitive to photodynamic chlorophyllin treatment and could be inactivated by a chlorophyllin concentration of 1 mg/L. So far, we have no explanation for this increased susceptibility that differs from other Gram-negatives such as *E. coli*. In contrast to *E. coli*, *S.* Typhimurium lacks a capsule [67]. For other species, like *K. pneumoniae*, it was shown that the capsule polysaccharide can mediate resistance to antimicrobial substances by limiting their interaction with membrane targets [68]. When Pereira et al. [69] investigated the photodynamic efficacy of cationic porphyrins they found that the chemical composition of external structures seems to have strong impact on the light-dependent inactivation of bacteria by photosensitizers. Hence, it may be that the envelope of *Salmonella* allows improved action of photosensitizing chlorophyllin but not necessarily an increased uptake as we could not detect any activity in darkness.

**Figure 7:**
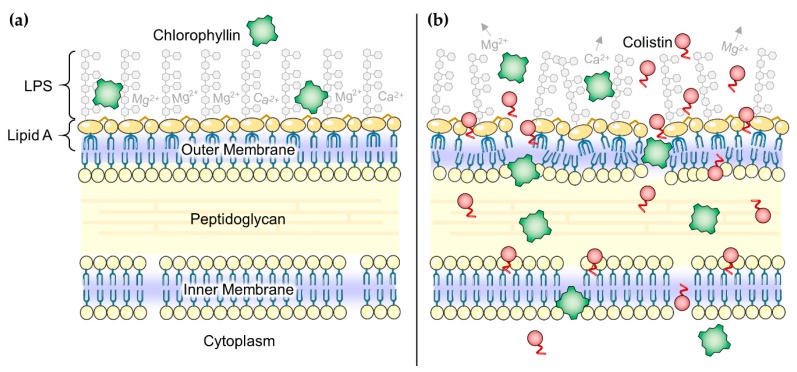
(**a**) Schematic illustration of the Gram-negative envelope. Chlorophyllin (green squares) is not able to pass the outer membrane; (**b**) In the presence of colistin (red circles) the outer membrane is disorganized, mainly by colistin’s effect to interact with lipopolysaccharides (LPS). Chlorophyllin can enter the bacterial cell through the disorganized membranes, realizing its antibacterial effect. Parts of the figure were drawn by using pictures from Servier Medical Art, licensed under a Creative Commons Attribution 3.0 Unported License (https://creativecommons.org/licenses/by/3.0/).

Gram-negative bacteria have an additional membrane layer (outer membrane) comprising glycolipid lipopolysaccharides (LPS) and phosphoglycerides as major components (Figure 7a). The outer membrane serves as a barrier to protect against toxic compounds, including antibiotics having targets other than this surface layer, and host innate immune molecules (e.g., cationic antimicrobial peptides) [70]. Indeed, some bacteria acquire antibiotic resistance by modifying the permeability of the outer membrane [71]. It is very likely that affecting the outer membrane of Gram-negatives makes them much more sensitive against bactericides, which otherwise are blocked. A recent study [72] described that the human complement system perforates this barrier through pore-formation by membrane attack complexes leading to treatability of infections caused by Gram-negatives with antibiotics that are considered ineffective. Other studies have shown the outer membrane as a target for destabilizing molecules [73]. Chemical destabilization can be achieved with various classes of substances, such as cationic detergents, chelators like ethylene-diamine-tetraacetic acid (EDTA), large cationic peptides, compound 48/80 (a polycationic polymer of p-methoxyphenethylmethylamine monomers) and others [62,74]. In this study, we used colistin to disturb the outer membrane permeability (Figure 7b) [75]. The possible observed additive effect of colistin and illumination on the proliferation of *E. coli* (and to a lower extent on S. Typhimurium) could not only be explained by delayed cell growth due to illumination as reported before [60]. To date, light-activation was only reported for tetracyclines [76] and the antitumor antibiotics ravidomycin and desacetylravidomycin [77], but this finding deserves further investigation of polymyxins.

Previous approaches on the combination treatment of destabilizing and antibacterial molecules to inactivate Gram-negatives were reported for chitosan, lactic acid, polyethyleneimine and polymyxin B nonapeptide. Chitosan, a product from deacetylated chitin of shrimps, was found to increase the permeability of the outer membrane significantly in acidic environment as shown using the fluorescent probe NPN [78]. The combination of chitosan with chlorophyllin-based photosensitization was able to reduce viable *Salmonella* effectively in the presence of light [79,80]. Lactic acid was also found to disrupt the outer membrane of *E. coli*, *P. aeruginosa*, and *S.* Typhimurium, but, same as chitosan, only at low pH [81,82]. Alakomi et al. [81] used lactic acid to sensitize Gram-negative bacteria for detergents. They proposed that exclusively the non-dissociated form has permeabilizing properties. In a further approach recombinant *Lactococcus lactis* was used to produce and secrete not only lactic acid but also heterologous antimicrobial peptides with activity against *E. coli* and *Salmonella* [83]. That the antibacterial efficacy of colistin is improved at low pH could also be demonstrated when a photoacid was combined with colistin treatment. In this approach the MIC of colistin decreased ~32 times for *P. aeruginosa* [84]. Nitzan et al. [85] investigated the effect of polymyxin nonapeptide (polymyxin B, similar to colistin) as a membrane-destabilizing agent with deuteroporphyrin and demonstrated effective photodynamic activity of the porphyrin in *E. coli* and *P. aeruginosa* in the presence of polymyxin B. Similar observations were noted in our study as well. Le Guern et al. [86] synthesized a peptide-coupled photosensitizer built of a cationic porphyrin and polymyxin B. They found an improved photodynamic efficiency against *P. aeruginosa* and *E. coli*. Confocal microscopy showed that the porphyrin-peptide selectively sticks to the cell walls.

Not all Gram-negative bacteria show the same sensitivity against substances weakening the outer membrane. Alakomi et al. [87] tested a combination of membrane-destabilizing agents (polyethyleneimine, EDTA and meso-2,3-dimercaptosuccinic acid) in combination with the biocide benzalkonium chloride. While strains of *Pseudomonas* and *Stenotrophomonas* became permeabilized in the presence of the agents (determined with NPN accumulation), strains of *Sinorhizobium* were found not to be affected. This indicates that the biochemistry of the LPS of the outer membrane seems to be very important for interaction with permeabilizers. We believe that further investigation of synergistic effects of chlorophyllin with different permeabilizers and in different strains is very promising to elucidate effective methods for inactivation of pathogenic bacteria.

Due to the light-dependency of photodynamic reactions, technical or medical applications of photosensitizers are restricted to exposed areas of action (e.g., wounds or surfaces), which can be illuminated. Chlorophyllin, in contrast, is not restricted to illumination to exert antibacterial properties but also shows activity in the darkness. For instance, according to our data, 0.25 µg/mL of colistin in combination with 10 mg/L chlorophyllin are sufficient to prevent the growth of *E. coli* in vitro. In addition, 0.25 µg/mL colistin in presence of 1 mg/L chlorophyllin inhibited the growth of *S.* Typhimurium. The underlying light-independent mechanisms are not yet understood. For possible medical applications, it is crucial to know the maximal concentration tolerated by the human body. Successful in vivo treatment of *Streptococcus septicemia* infections with chlorophyllin was firstly reported by Gruskin in 1940 [88]. However, the interest in chlorophyll as an antibiotic and as a therapeutic agent in general appears to have dwindled as conventional antibiotics arose; there is only sporadic reporting of studies in this field for almost five decades. Only since the year 2000 has chlorophyll and its derivatives caught attention again as is reflected in a higher number of publications. The few and vague information available [88,89] on intravenously use of chlorophyll derivatives indicates that it is—at least theoretically—possible to achieve the mandatory concentrations in a patient without significant side effects.

### 3.2. Overcoming Colistin Resistance of E. coli Using Colistin in Combination with Chlorophyllin

**Figure 8:**
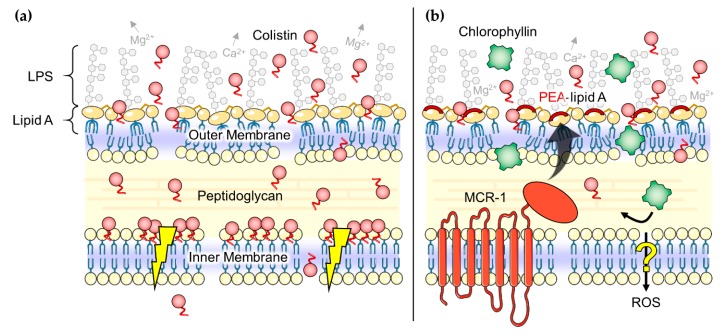
(**a**) Postulated effects of colistin (red circles) on Gram-negative bacteria. Colistin molecules disorganize the outer membrane and may somehow disrupt the physical integrity of the inner membrane (flash symbols) finally leading to cell death [75]; (**b**) Hypothesized ‘door opener’ effect of colistin for photodynamic treatment with chlorophyllin (green squares). The fatty-acyl tail is still able to perturb the outer membrane but effects on the inner membrane seems to be impaired by the protein MCR-1. Our data suggests that MCR-1 inhibits the uptake of chlorophyllin over the inner membrane (no inactivation of bacteria in the dark) but might allow the passing of chlorophyllin’s photodegradation products such as ROS (inactivation in light). LPS: lipopolysaccharides, PEA: phosphoethanolamine, ROS: reactive oxygen species. Parts of the figure were drawn by using pictures from Servier Medical Art, licensed under a Creative Commons Attribution 3.0 Unported License (https://creativecommons.org/licenses/by/3.0/).

Synergistic effects of colistin in combination with other antibiotics, such as rifampicin, tigecycline, eravacycline, clarithromycin, minocycline, novobiocin, or fusidic acid against colistin- or carbapenem-resistant Gram-negative pathogens have been reported before [90,91,92,93,94,95]. In addition, other combinations, for example, of colistin with the membrane-perturbing phytoalexin resveratrol showed promising results [96,97]. It is indicated that colistin has properties to increase antibiotic activity in *mcr-1*-positive bacteria, but this ability is not well understood so far [95,98]. MacNair et al. [95] suggested that the fatty-acyl tail of colistin (Figure 1a, yellow), an important factor for polymyxin toxicity [99], plays a two-edged role in this mechanism: on the one hand cell lysis and self-promoted uptake of colistin seems to be impaired by the protein MCR-1, on the other hand the fatty-acyl tail is still able to disorganize the outer membrane (Figure 8b) [95,100,101]. Thus, colistin can act as a door opener for other antimicrobial substances such as chlorophyllin, even for the inactivation of *mcr-1*-positive *E. coli* cells. In this study, we could demonstrate that chlorophyllin in combination with therapeutic colistin is effective to kill *mcr-1*-positive *E. coli* cells, but the effect was limited to the photodynamic activity of chlorophyllin [60]. Chlorophyllin was not able to inhibit bacterial growth in the absence of light. To what extent the presence of MCR-1 protein can affect chlorophyllin’s dark-active mechanism of action has to be explored in future studies. There are some hints that only the photodegradation products of chlorophyllin such as ROS, but not chlorophyllin itself, can pass the inner membrane in presence of MCR-1 (Figure 8b). In accordance with our results, Pourhajibagher et al. [102] found a reinforcing effect of photodynamic treatment with toluidine blue O in combination with colistin against pandrug-resistant *A. baumannii*.

Altogether, the door opener ability of colistin opens up new possibilities to control *mcr-1*-expressing pathogens with colistin combination therapies. Ironically, the susceptibility of colistin-‘resistant’ bacteria to colistin—albeit not lethal—may be an Achilles heel for a problematic resistance mechanism in Gram-negative bacteria (Table 1) [103]. Most recently, the ninth *mcr* homologue *mcr-9* has been identified in *Salmonella* [25]. It remains to be investigated whether all *mcr* variants are susceptible to colistin combination therapy.

## 4. Materials and Methods 

### 4.1. Bacteria Strains and Growth

Experiments were performed with *E. coli* DH5α (Invitrogen, Carlsbad, CA, USA) and *S. enterica* subsp. *enterica*, serovar Typhimurium Ames9274 (laboratory stock). The bacteria were grown in LB medium overnight in an incubator at 37 °C and 150 rpm. Prior to the experiments, cell concentration was determined by multiple measurements of absorbance at 590 nm and set to OD_590_ = 0.1 before cells were diluted in LB medium as required. During the experiments, cells were grown on/in LB at 37 °C.

For the generation of a colistin-resistant mutant, *E. coli* DH5α was transformed with the pGDP2:*mcr-1* plasmid (kindly provided by Eric D. Brown, McMaster University, Hamilton, ON, Canada). Antimicrobial susceptibility of the strains against colistin was tested by using the MICRONAUT MIC-Strip colistin (Merlin Diagnostika, Bornheim, Germany) and broth microdilution, as recommended by the Clinical and Laboratory Standards Institute (CLSI) and the European Committee on Antimicrobial Susceptibility Testing (EUCAST) [104,105].

### 4.2. Preparation of Chlorophyllin

Chlorophyllin preparation from frozen spinach was performed as described before [60]. The concentration of chlorophyllin was determined spectrophotometrically using the formula of Ziegler and Egle [106]. Aliquots were stored in darkness at −20 °C prior to use. The collected extract was dried and the chlorophyllin powder was dissolved in LB medium to a working solution of 50 mg/L.

### 4.3. Growth Experiments

Colistin sulfate salt was purchased from Sigma-Aldrich (Steinheim, Germany) and dissolved in LB to a working solution of 50 mg/mL.

For suspension cultures, cells were directly incubated in 1 mL acrylic photometer cuvettes (Sarstedt, Nümbrecht, Germany) after adjusting different chlorophyllin (0.0, 0.25, 0.50, 1.0, 2.5, 5.0, 10.0, 15.0, 20.0, and 25.0 mg/L) and colistin concentrations (0.0, 0.005, 0.010, 0.025, 0.050, 0.100, 0.250, 0.500, 1.000, and 2.500 μg/mL) from stock solutions. The initial cell density was set to OD_590_ = 0.1 (corresponding to 0.8 × 10^8^ cfu/mL). Cell-free medium with corresponding chlorophyllin and colistin concentrations served as blanks. Cuvettes were kept in their original styrofoam boxes, which were placed into sterile plastic bags to avoid contamination during light exposure. Two identical boxes were prepared for illumination and for dark control (wrapped in aluminum foil). Both boxes were incubated on a shaker at 37 °C inside an incubator equipped with a LED grow light PRAKASA 300 W (Green Tech Direct Ltd., Harrow, Middlesex, UK). Distance to samples was adjusted to achieve a photon flux of 560 µE/(s × m²) which corresponds to a light intensity of 12 mW/cm^2^. Cell growth was determined photometrically at 590 nm (OD_590_) prior to exposure (0 min), and subsequently after defined time intervals. During the measurements, exposure to light was interrupted until all samples were measured.

For the CFU assay, samples were prepared as described before [60]. Before incubation as well as in subsequent defined time-intervals each 5 µL of each cell sample were transferred on agar plates. After overnight incubation at 37 °C, plates were inspected for colony-formation. Untreated cell samples served as controls. Corresponding spots where cell solution was added were classified as follows: colony formation same as in controls: no effect; colonies obviously smaller compared to controls: inhibition; only max. two single small spots visible: strong inhibition; no colony formation: complete inhibition.

All experiments were prepared in triplicates (n = 3) and repeated at least thrice.

### 4.4. Determination of Minimal Inhibitory Concentrations (MICs)

MICs of chlorophyllin and colistin, and of their combinations were determined as described elsewhere [107]. Sterile 48-well flat bottom microtiter plates (Thermo Fisher Scientific, Waltham, MA, USA) were filled with 500 μL of LB medium and different concentrations of chlorophyllin (for *E. coli*: 0.31–20 mg/L, for *Salmonella*: 0.03–2 mg/L) and colistin (0.06–1 µg/mL, for *E. coli* DH5α pGDP2:mcr-1: 0.5–8 µg/mL). Bacteria (*E. coli* DH5α, *E. coli* DH5α pGDP2:mcr-1 or *S.* Typhimurium Ames9274) were inoculated at a titer of 5 × 10^6^ cfu/mL. Two sets of microtiter plates were prepared. One set was incubated under illumination (12 mW/cm^2^) and other set was wrapped with aluminum foil for dark incubation. After incubation at 37 °C for 24 h, the MIC was determined as the last dilution at which no turbidity was observed. The MIC tests were repeated thrice.

For the additional determination of growth kinetics, 5 μL samples from each well were transferred into a new 96-well microtiter plate (Thermo Fisher Scientific, Waltham, MA, USA) containing 200 µL of sterile LB medium in each well. The plates were optically analyzed after further incubation at 37 °C for 24 h.

### 4.5. NPN Uptake Assay

Outer membrane permeability was determined using the fluorescent probe 1-N-phenylnaphthylamine (NPN) as described [108]. In contrast to the published protocol, bacteria were resuspended in phosphate-buffered saline and OD_600_ values between 0.005 and 0.3 were applied. All experiments were carried out at least in triplicates (biological replicates) with two technical replicates (duplicates). NPN was purchased from Sigma-Aldrich and used in final concentrations between 0.5 µM and 10 µM. Measurement were carried out using a Tecan Infinite M Nano+ plate reader (Tecan Group, Männedorf, Switzerland) with Nunc MicroWell 96-well flat bottom microtiter plates (Thermo Fisher Scientific, Waltham, MA, USA).

### 4.6. Fluorescence Microscopy

Visualization of chlorophyllin uptake in *E. coli* was performed using the Biozero BZ-8000 digital fluorescence microscope (Keyence, Osaka, Japan). Fluorescence images were taken from liquid cultures with a 100× oil immersion objective, an excitation wavelength of 450–490 nm and an emission cut-off at 510 nm.

### 4.7. Data Evaluation and Statistics

After subtraction of the optical densities of corresponding cell-free blanks from those of the samples, mean values as well as standard deviation of triplicates were calculated and are shown as graphs.

Differences between samples and corresponding controls were analyzed with an independent-samples *t*-test using the IBM SPSS Statistics 23.0 software (IBM Deutschland GmbH, Ehningen, Germany). *p* < 0.05 was considered as statistically significant.

Synergistic effects of colistin/chlorophyllin combinations were assessed using the Combenefit software (Cancer Research UK, Cambridge, UK) [109]. Available concentrations of both substances and dose response information (normalized to control conditions) were entered into the program. The software automatically analyzed the numeric data employing the Loewe additivity model to identify synergism, antagonism or additive in drug combinations.

## 5. Conclusions

In this study, we demonstrated that chlorophyllin successfully inhibited the growth of Gram-negative bacteria (*E. coli* and *S.* Typhimurium) in the presence of colistin (Table 1). For the proof of concept, we used sub-toxic colistin concentrations. In clinical context, higher colistin concentrations are recommended to prevent spreading of resistance. Chlorophyllin’s effect on cells was more pronounced under illumination, confirming the photosensitizing activity of chlorophyllin. *Salmonella* was more susceptible to chlorophyllin than *E. coli*. This suggests that the chemical composition of the outer membrane can have a strong impact on photosensitization efficacy. Furthermore, a combined effect of colistin and chlorophyllin was observed against *mcr-1*-positive *E. coli* cells indicating a potential application of chlorophyllin combination therapy to treat drug-resistant pathogens. However, even if more pathogenic strains and drug combinations have to be tested in future, we speculate that, if applied together with substances weakening the outer membrane, chlorophyllin may become an effective antimicrobial substance also for the control of Gram-negative bacteria.

## Figures and Tables

**Table 1:** Chlorophyllin-based inactivation of Gram-negative model strains. Strategies to inhibit cell growth in light (bright column) or darkness (grey column).

Strain	Illuminated with 12 mW/cm^2^	Dark-Incubated
*E. coli* DH5α	5 mg/L chlorophyllin + 0.125 µg/mL colistin or 2.5 mg/L chlorophyllin + 0.25 µg/mL colistin or 1.25 mg/L chlorophyllin + 0.5 µg/mL colistin	10 mg/L chlorophyllin + 0.25 µg/mL colistin or 2.5 mg/L chlorophyllin + 0.5 µg/mL colistin
*E. coli* DH5α pGDP2:*mcr-1*	10 mg/L chlorophyllin + 2 mg/L colistin or 5 mg/L chlorophyllin + 4 µg/mL colistin	no inactivation at low chlorophyllin concentrations; observed inactivation with 20 mg/L chlorophyllin + 4 µg/mL colistin
*S.* Typhimurium Ames9274	1 mg/L chlorophyllin ^1^ or 0.063 chlorophyllin + 0.25 µg/mL colistin or 0.125 chlorophyllin + 0.5 µg/mL colistin	1 mg/L chlorophyllin + 0.25 µg/mL colistin or 0.125 mg/L chlorophyllin + 0.5 µg/mL colistin

^1^*Salmonella* showed increased susceptibility to photodynamic chlorophyllin treatment.

## Supplementary Materials

The following are available online at https://www.mdpi.com/2079-6382/8/4/158/s1, Figure S1: Possible additive effect of colistin and light on the proliferation rate of *E. coli* DH5α and *S.* Typhimurium; Figure S2: Growth kinetic of *E. coli* DH5α in the presence of chlorophyllin/colistin concentrations; Figure S3: Chlorophyllin uptake into *E. coli*, *S.* Typhimurium and *E. coli* pGDP2:*mcr-1* in the presence of colistin; Table S1: Effects of different colistin concentrations on the growth of *E. coli* DH5α.
