# Supplementary material for: Using Colistin as a Trojan Horse: Inactivation of Gram-Negative Bacteria with Chlorophyllin
Source: Antibiotics (Basel). 2019 Sep 20;8(4):158. doi: 10.3390/antibiotics8040158 (PMC6963628; doi:10.3390/antibiotics8040158)
Supplement: Supplementary file 1 [file antibiotics-08-00158-s001.pdf]

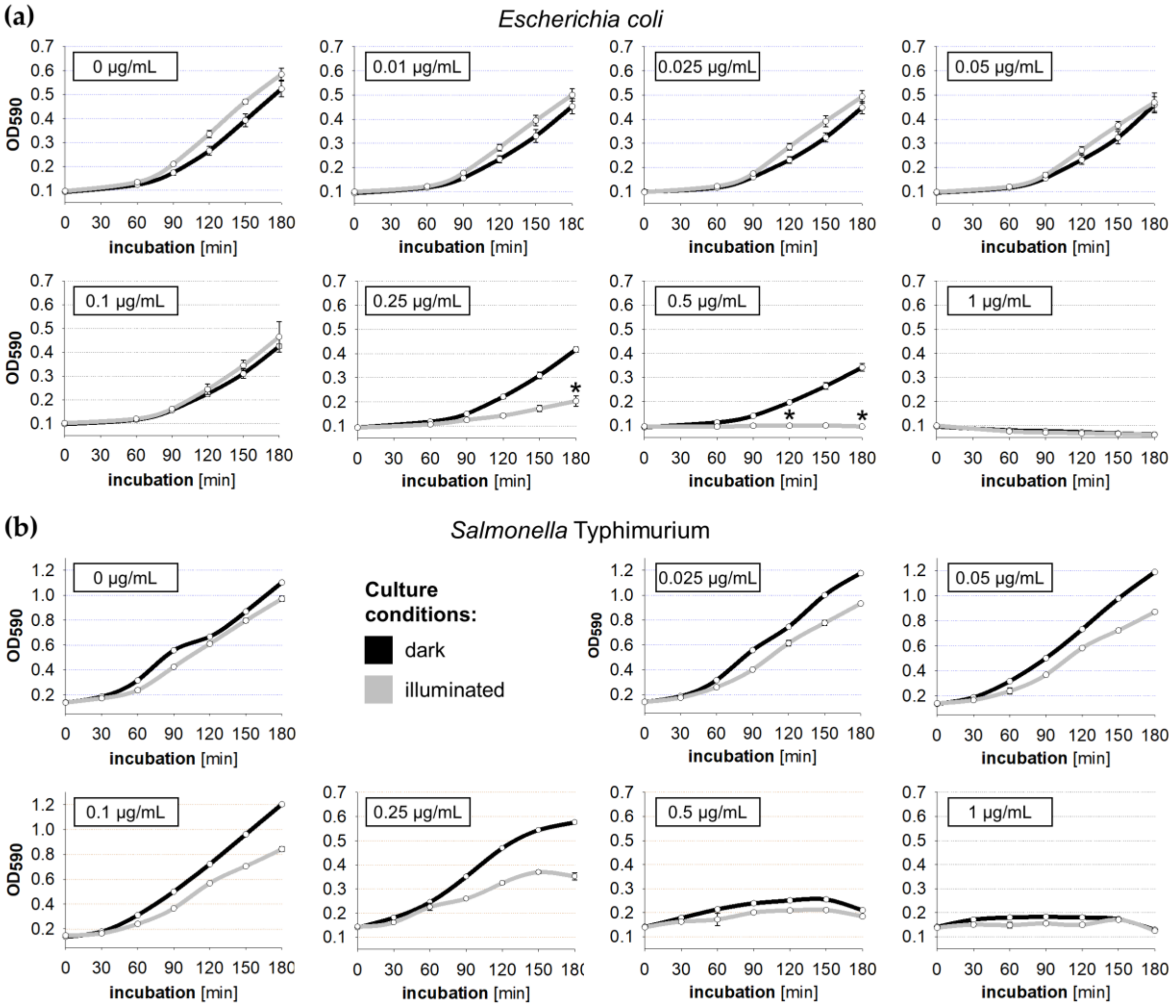

Figure S1. Possible additive effect of colistin and light on the proliferation rate of (**a**) *E. coli* DH5α and (**b**) *S.* Typhimurium. LB was supplemented with different colistin concentrations. Cells grew either illuminated with 12 mW/cm^2 (grey lines) or protected from light (black lines). Depicted are measured values (circles) and fitted curves (lines) ± standard deviations (n = 3) showing one representative of three independent experiments. *: $p < 0.05$ vs. not-illuminated-free samples.

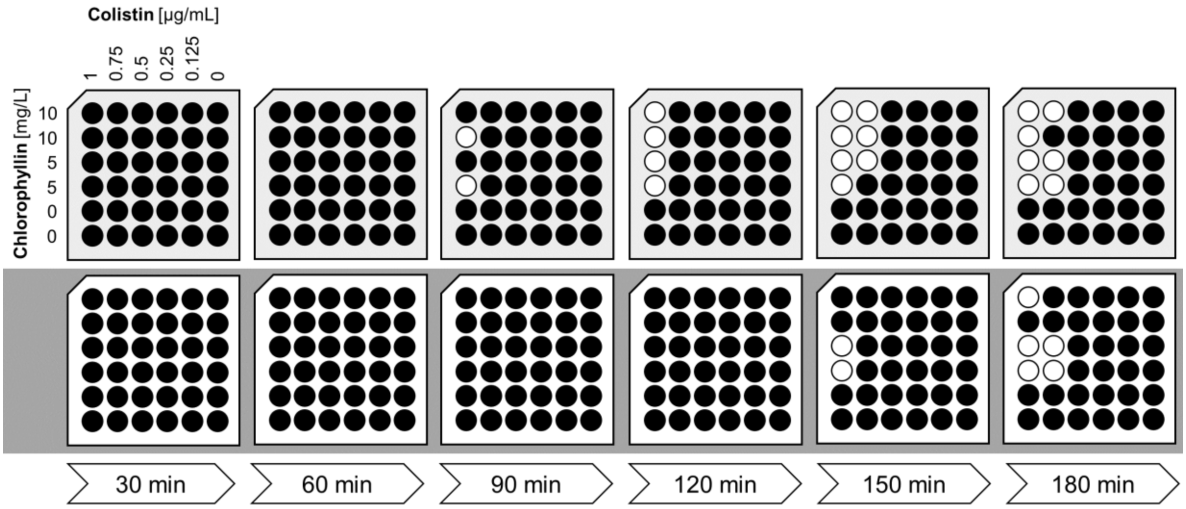

Figure S2. Growth kinetic of *E. coli* DH5α in the presence of chlorophyllin/colistin concentrations. Liquid cultures containing chlorophyllin and/or colistin were exposed to light (upper row) or were protected from light (lower row; grey). In 30-minute intervals, 5 µL samples of were transferred into new 48 well plates with LB medium without supplementations. Cell growth was checked after further 24 h incubation estimating the turbidity of the medium inside the wells. Black circles indicate turbidity (=living cells), white circles no turbidity (=no living cells).

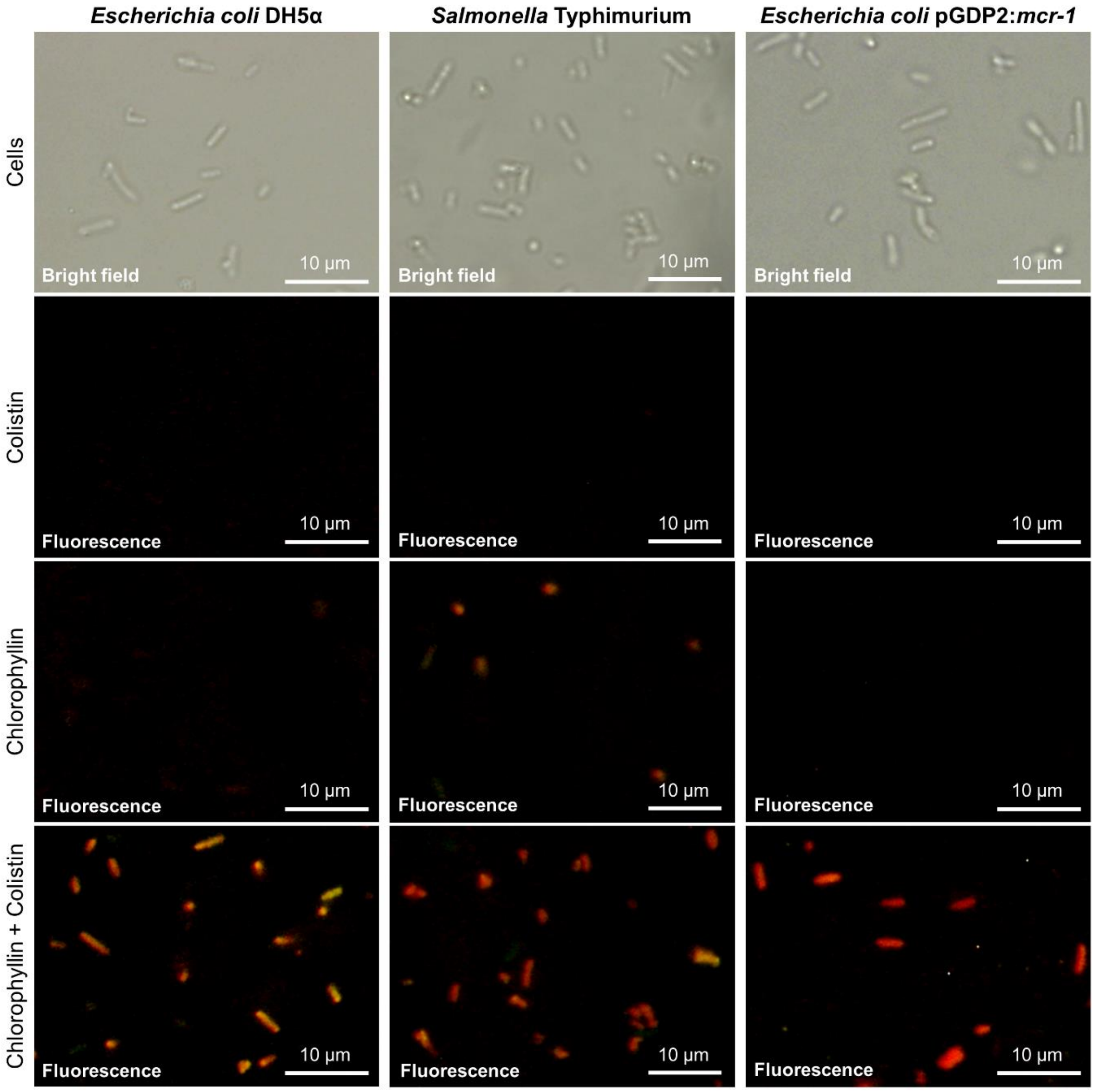

Figure S3. Chlorophyllin uptake into *E. coli*, *S.* Typhimurium and *E. coli* pGDP2:*mcr-1* in the presence of colistin. Samples were taken from liquid cultures after 24 h of incubation in darkness and microscopically analyzed in bright field (first row) and under blue light fluorescence. Red fluorescence is emitted from chlorophyllin inside the cells. Scale bars: 10 µm.

Table S1. Effects of different colistin concentrations on the growth of *E. coli* DH5α. Given are OD_{590} values ± standard deviations (n = 3) showing one representative of three independent experiments.

**Time**	**Colistin concentrations [µg/mL]**									
**[min]**	**0.000**	**0.005**	**0.010**	**0.015**	**0.050**	**0.100**	**0.250**	**0.500**	**1.000**	**2.500**
**0**	0.097 ±0.001	0.098 ±0.003	0.099 ±0.002	0.097 ±0.001	0.098 ±0.001	0.102 ±0.003	0.093 ±0.002	0.097 ±0.002	0.100 ±0.001	0.099 ±0.001
**60**	0.134 ±0.004	0.124 ±0.004	0.121 ±0.002	0.122 ±0.002	0.120 ±0.001	0.119 ±0.003	0.107 ±0.003	0.095 ±0.002	0.076 ±0.001	0.078 ±0.001
**90**	0.210 ±0.007	0.186 ±0.008	0.177 ±0.005	0.174 ±0.006	0.168 ±0.010	0.161 ±0.006	0.125 ±0.003	0.099 ±0.001	0.071 ±0.001	0.075 ±0.001
**120**	0.335 ±0.015	0.302 ±0.011	0.282 ±0.013	0.285 ±0.014	0.271 ±0.015	0.244 ±0.021	0.143 ±0.008	0.099 ±0.003	0.068 ±0.000	0.070 ±0.001
**150**	0.470 ±0.008	0.414 ±0.015	0.394 ±0.022	0.391 ±0.023	0.374 ±0.017	0.344 ±0.021	0.172 ±0.013	0.100 ±0.005	0.066 ±0.001	0.067 ±0.001
**180**	0.584 ±0.025	0.529 ±0.024	0.500 ±0.025	0.493 ±0.026	0.470 ±0.038	0.465 ±0.064	0.203 ±0.021	0.096 ±0.006	0.062 ±0.001	0.063 ±0.001
